# The Effects of Age, Priming, and Working Memory on Decision-Making

**DOI:** 10.3390/ijerph13010119

**Published:** 2016-01-11

**Authors:** Meagan Wood, Sheila Black, Ansley Gilpin

**Affiliations:** Psychology Department, University of Alabama, P.O. Box 870348, Tuscaloosa, AL 35487, USA; mmwood2@crimson.ua.edu (M.W.); agilpin@ua.edu (A.G.)

**Keywords:** decision-making, working memory, older adults

## Abstract

In the current study, we examined the effects of priming and personality on risky decision-making while playing the Game of Dice Task (GDT). In the GDT, participants decide how risky they wish to be on each trial. In this particular study prior to playing the GDT, participants were randomly assigned to one of three priming conditions: Risk-Aversive, Risk-Seeking, or Control. In the Risk-Seeking condition, a fictional character benefitted from risky behavior while in the Risk-Aversive condition, a fictional character benefitted from exercising caution. Although not explicitly stated in the instructions, participants need to make “safe” rather than risky choices to optimize performance on the GDT. Participants were also given Daneman and Carpenter’s assessment of working memory task. Interestingly, although older adults self-reported being more cautious than younger adults on the Domain Specific Risk Attitude scale (DOSPERT), older adults made riskier decisions than younger adults on the GDT. However, after controlling for working memory, the age differences on the GDT became insignificant, indicating that working memory mediated the relation between age and risky decisions on the GDT.

## 1. Introduction

Older adults are often confronted with risk when making important decisions about their lives. For example, they are confronted with risk in making decisions about insurance policies, elective medical procedures, *etc*. The ability to accurately assess risk involves cognitive resources, because one needs to engage in an extensive cost/benefit analysis assessment to make the best decision when risk is involved. Due to age-related changes in cognitive resources older adults might not appreciate the level of risk associated with some ventures such as purchasing a product through a telemarketer, and as a result might make hasty and possibly costly decisions. The goal of the current study is to examine age differences in risky decision-making as a function of age, personality, attitude, and working memory processes.

One popular way of measuring age differences in risky decision-making is through gambling paradigms such as the Iowa Gambling Task (IGT). However, the IGT is associated with a considerable amount of ambiguity because participants are not told any rules and must implicitly learn them from experience. Participants are presented with four decks (some are more advantageous than others) and are told to try to gain as much money as possible. It is thought that, initially, participants make judgments implicitly, because they are implicitly but not explicitly aware of the outcomes associated with each deck of cards. However, after a number of trials, young adults at least become aware of the outcomes associated with each deck of cards. Several studies have found age differences in the IGT, but there has been no consensus among researchers about the source of the age differences, given that the IGT measures both implicit and explicit abilities [1].

**Figure 1:**
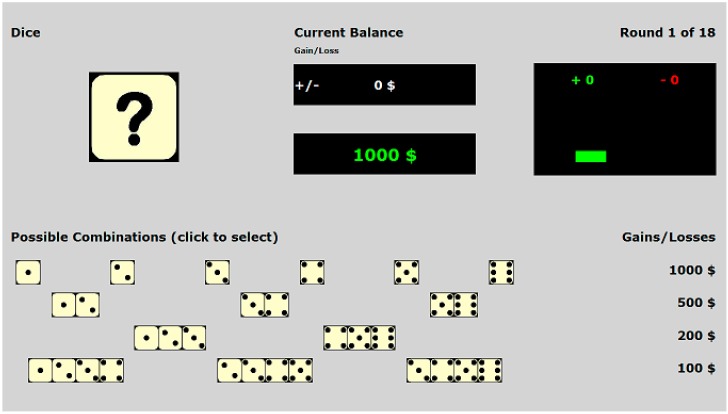
Screenshot of Game of Dice Task (GDT).

A number of new gambling tasks have emerged that provide a way for assessing risk-taking when information is readily presented for the decision-maker. One example of a gambling task that is more straightforward than the IGT is the Game of Dice (GDT). The Game of Dice Task (GDT) is a probability-based gambling task that requires participants to guess what number(s) will come up when they “roll the dice”. Figure 1 provides a screen shot of the options presented to participants. In each trial, the participant is presented with four rows. Each row is associated with a certain level of risk. The top row is the riskiest and the bottom row is the safest. To explain further, in the bottom row, the dice are presented in groups of four. If the participant decides he/she wants to choose one of the options presented in the bottom and safest row, the participant will win money as long as one of the numbers among the four dice comes up when he/she “rolls the dice”. On the other hand, in the top row, each option consists of one die. If the participant chooses an option from this row, he/she will only win money if the selected numbers come up when the die is rolled. As one might expect, the amount of money that can be gained varies as a function of risk. Thus, the participant can choose to hedge his/her bets on just one number and win more money (*i.e.*, $1000.) or the participant can decide to be safer and place the bet so that he/she will win less money ($100). The participant’s wins and losses are displayed throughout the game. The game is designed so that making risky choices on a consistent basis will result in participants’ losing money. The GDT has been touted as a gambling task that is a purer measurement of risk than the IGT, because it does not contain the ambiguity inherent in the IGT.

### 1.1. Cognitive Resources and the GDT

Although information is presented in a more explicit way in the GDT than in the IGT, it still requires the engagement of higher order reasoning skills to maximize profit and minimize risk. In fact, Brand and colleagues have argued that working memory/executive resources might be more important for the GDT than for the IGT because the IGT relies partially on implicit learning whereas the GDT relies primarily on explicit learning [1,2,3,4,5,6,7].

It may be somewhat surprising that the GDT relies on cognitive resources when the information is presented ostensibly in a straightforward way. There are two reasons that the GDT may rely on working memory resources. The first reason focuses on the relation between working memory and the ability to fully understand information that is not explicitly stated. Although the GDT is more explicit than the IGT, much of the information is implied rather than explicitly stated. The ability to deduce information that is not explicitly stated is highly dependent on working memory [8,9]. Further, the GDT relies on a certain level of numeracy, as it is necessary to calculate the probabilities of wins and losses (based on the instructions) to fully understand the consequences of choices. This ability to make predictions based on probability calculations is dependent on working memory [10].

The second reason that we believe that the GDT is reliant on working memory is that optimizing performance on the GDT relies on profiting from feedback. In order to benefit from feedback, a participant would need to monitor wins and losses while playing the game and be able to shift strategy to maximize profit. All of the aforementioned cognitive functions require working memory, particularly the executive component of working memory.

### 1.2. Working Memory, Age, and GDT

Based on the plethora of studies indicating age differences in working memory [11,12,13,14], and the evidence that the GDT is dependent on executive processing and working memory [3,4,15], we predicted that there would be age differences in the GDT. As mentioned earlier, working memory resources would be necessary to make the required inferences about the risks associated with certain options. Due to age-related changes in working memory, older adults have difficulty making inferences when information is not explicitly stated [5,8,12,16].

Working memory, as originally conceptualized by Baddeley [17] involves the ability to store and manipulate information. More recently, Just and Carpenter [9] conceptualized working memory as activation that is limited in capacity. They argue that activation available for working memory processes differ among individuals which ultimately has an impact on cognitive resources available for the storage and manipulation of to-be-comprehended information. In the current study, older adults will have to make inferences about the probabilities or risks associated with each of their choices, because the standard instructions only mentions various combinations of die; there is no mention of the risks associated with each choice. We would argue that participants would have to tax working memory to properly assess the risk associated with each of their choices. Participants receive the following instructions prior to playing the GDT:

“Welcome to the Game of Dice Task. In this task, you are going to throw a virtual dice 18 times. Before each throw, you will be able to bet on the outcome by selecting a single number (e.g., ‘3’) or combinations of two to four numbers (e.g., ‘1-2-3’). You are given a starting capital of $1000. Your job is to maximize this capital within 18 throws of the dice. Good luck”!

On the surface, the aforementioned sentences may seem easy to comprehend; however, comprehending the full meaning of the aforementioned sentences may not be as easy as it seems. Participants would have to unpack the meaning behind those three sentences to understand that the probability of winning money increases as the number of dice associated with an option increases. Actually coming to the realization that the probability of winning is tied to the number of dice associated with a particular option is a multi-step process. First, participants would have to retrieve information from Long-term-memory related to the configuration of a typical die (e.g., remembering that a single die has six sides). Second, they would have to retrieve probability-related information from long-term-memory to realize that when a die is thrown, there is a one in six chance that a given number will come up; however, if two dice are thrown there is a two and six probability and so forth. Again, participants would have to realize that their probability of winning increases as the number of dice associated with a particular option increases. After retrieving the aforementioned information from long term memory, they would need to apply the information to the rows of dice displayed on the computer screen, as depicted in Figure 1. A number of studies have pointed out that the integration process is particularly dependent on working memory [8,9,18]. We would argue that fully appreciating the risk associated with the various choices in the GDT requires a number of working memory computations [19].

Even if participants are not able to comprehend the instructions well enough to play the GDT optimally, this game still provides another opportunity to develop a strategy to maximize performance. Participants can monitor the computer-generated feedback as they play the game. For example, the GDT is designed so that the participant can actually track gains and losses on the computer screen while playing the game. However, participants would have to employ working memory/executive resources to benefit from feedback and to shift strategies as a result of the feedback. Due to age-related changes in working memory and executive processes, a number of studies indicate that older adults are not as adept as younger adults in initiating strategies without environmental support due to age-related changes in cognitive resources [20,21,22]. Thus, we predicted that older adults would be at a disadvantage in their ability to formulate strategies based on feedback, and due to age-related changes in flexibility, older adults would have more difficulty than their younger counterparts in switching strategies based on feedback [1,13].

For example, working memory also plays an important role in the strategizing necessary to produce optimal performance on the GDT. Due to age-related changes in working memory and executive processes, a number of studies indicate that older adults are not as adept as younger adults in initiating strategies without environmental support due to age-related changes in cognitive resources [20,21,22]. Thus, we predicted that older adults would be at a disadvantage in their ability to formulate strategies based on feedback, and due to age-related changes in flexibility, older adults would have more difficulty than their younger counterparts in switching strategies based on feedback [1,13].

### 1.3. Personality and the GDT

In addition to cognitive processes affecting risky decision-making, other individual characteristics such as personality may have an effect on risk-taking. We were interested in determining the extent to which personality traits such as openness to experience would directly influence risk-taking on the GDT. Various dimensions of the Big Five Personality Test have been shown to be correlated with risky decision-making. Specifically, high scores in the dimensions of extraversion and openness and low scores in neuroticism have been correlated with risky decision-making. Nicholson and colleagues, in particular, found that high scores on the Extraversion and Openness dimensions of the Big Five Personality Inventory were associated with risk-taking [23]. More recently, Brand and Altstötter-Gleich, [24] conducted research which indicates that perfectionism affects behavior. Specifically, there was evidence that participants that were leery of making mistakes performed better on the GDT than individuals who were less concerned with errors. Although research has shown that younger individuals high in openness and extraversion were more likely to engage in risk-taking than individuals who were not high on those dimensions, very little research has examined the impact of personality on risk-taking among older adults. Nevertheless, the few studies that have been conducted have found that older adults who were high in openness were more apt to be risk-takers than older adults who were low on that dimension [25]. Thus, in the current study, we predicted that extraversion and openness to experience would be the two personality dimensions of the Big Five most predictive of risk-taking.

In addition to personality influencing risk-taking on the GDT, we were interested in examining the impact of preexisting attitudes on risk-taking. Previous research has indicated that preexisting attitudes also affect the extent to which participants made risky choices across various experiments [26,27]. However, this past research mainly focused on young adults. Given that older adults are often in situations in which they are forced to assess risk in making decisions (deciding on financial investments or selecting the appropriate insurance policy), we wanted to examine the effects of preexisting attitudes on their decisions. Would a cautious attitude for example, protect older adults from making unwise risky decisions on the GDT? Because we also speculated that preexisting attitudes could potentially influence decision-making, participants were presented with the Domain Specific Risk Attitude scale (DOSPERT). The DOSPERT is a scale that assesses one’s propensity to take risk and one’s past risky behavior. We surmised that one’s perception of risk and one’s past risky behavior may affect the degree of risk-taking on the GDT.

There are a number of other factors besides preexisting personality traits and attitudes that affect decision-making and specifically, the propensity to take risk. Interestingly, sometimes information about tangentially related situations precedes the decision-making process. In many instances, the earlier exposure to the risk-related scenario should have no bearing on the current decision. However, exposure to risk-related information may subconsciously influence decision making through category accessibility. For example, a shopper may very seldom purchase a lottery ticket. However, she may hear about an acquaintance winning money by engaging in risky behavior. The exposure to the risk-taking scenario enhances the accessibility of concepts related to risk-taking. Because concepts associated with risk and adventure, are more accessible because of the earlier exposure, the shopper is now more willing to engage in risk-taking and thus buys a lottery ticket.

The current study was designed such that the GDT would be preceded by a prime scenario. Our prediction was that factors such as personality and preexisting attitudes would not only have a direct impact on risk-taking but that these factors would also *moderate the strength of the prime*.

### 1.4. Risky Primes and the GDT

There is growing research which indicates that category accessibility also affects decision-making. This is important because all of us are randomly presented with information that could influence decision-making, even in instances in which the information is not directly related to the decision-making task. It is conceivable that in real-world settings that people’s risk propensity may change based on accessible information at the time of decision. Increasingly, behavioral decision researchers realize that decisions are not made based solely on the structural properties or probabilities associated with a choice. Instead, decisions are ultimately influenced by factors such as semantic accessibility and other contextual factors [28]. For example, Kusev *et al.*, and other researchers [28,29,30,31] have provided compelling evidence that the probability of making risky decisions is determined in part by the availability of information during the decision-making process. Thus, addition to focusing on personality characteristic associated with risky decision-making, we focused on the way in which category accessibility could affect behavior. We manipulated category accessibility through a priming manipulation. For example, a person may be reluctant to invest in the stock market, after hearing of a friend’s loss of resources in a risky situation. We were interested in examining the effects of contextual variables with respect to the GDT. Although there have been studies that have examined the impact of contextual primes in other decision-making paradigms, to our knowledge, no published research has examined contextual variables with respect to the GDT.

The classic study focusing on the impact of category accessibility and subsequent judgments was conducted by Higgins, Rholes, and Jones, [32]. In the Higgins *et al.*, study, participants were presented with vignettes in which the main character could be described in a positive or negative way depending on one’s perspective. The researchers manipulated the way in which participants perceived the main character via a priming task. On some occasions, in an ostensibly unrelated task participants were primed with a positively valenced word such as “adventurous” and on other occasions, individuals were primed with a negatively valenced word such as “reckless”. The evidence indicated that participants primed with the word ”adventurous” judged the main character in a more favorable light than those who were primed with the word “reckless”. Higgins *et al.*, attributed their results to category accessibility.

Recently, the findings of the Higgins *et al.*, study [32] have been replicated with older adults [33]. In a series of studies, Hess and colleagues [34,35] have found that older adults are more likely to be influenced by priming effects than younger adults [33]. Age differences appeared to be due to younger adults being more aware of the prime’s influence and being more likely to employ the appropriate corrections to counteract the effects of the prime than older adults. That is, younger adults were more likely to be aware of the possible biases associated with the prime and were more likely to make an effort to suppress the effects of the prime. In the current study, we hypothesized that primes would affect risky decision-making in the GDT through category accessibility. As indicated earlier, a number of studies have shown that primes can affect decision-making [29,30,31].

In the current study, participants were assigned to one of three conditions: Risk-Seeking, Risk-Averse, and Neutral. In the Risk-Seeking condition, participants were presented with a scenario in which the main character was rewarded for risky behavior. In the Risk-Averse condition, participants were presented with a scenario in which risky behavior resulted in an undesirable outcome. Finally, in the neutral condition, participants did not receive a scenario prior to the gambling task.

We had three goals in conducting this study. First, we were interested in following up on the study conducted by Brand and Schiebener [1]. To our knowledge, only one study has been conducted thus far focusing on age differences in risky decision-making using the GDT [1]. We were interested in determining if Brand and Schiebener’s study could be replicated in another laboratory. We were also interested in extending Brand’s work to examine the possibility that contextual primes influenced the extent to which younger and older adults engaged in risk-taking. Finally, we were also interested in exploring the possibility that individual differences among people in each group would moderate the effects of the prime. That is, would individuals who were already predisposed to taking risks (*i.e.*, due to preexisting personality traits or attitudes) be more influenced by a risky prime than individuals less predisposed to take risks?

As mentioned earlier, our study was very similar to the Brand and Schiebener’s study [1]. In keeping with Brand and Schiebener’s study, we used the GDT to measure risky decision-making. However, we modified Brand and Schiebener’s study in several important ways. First, we assessed personality characteristics through the Big Five Personality Inventory to determine if personality characteristics affected the propensity to take a risk. We also examined the relation between preexisting attitudes toward risk and propensity to take risks in the GDT. Finally, we also examined the extent to which age differences in working memory might mediate the relation between age and risky choices in the GDT.

We made several predictions for the current study based on past research. First, based on the research examining the effects of a prime on decision-making, we predicted that the primes would affect participants’ risk propensity on the game of dice task. Based on the research examining age differences in decision-making and social judgments, we predicted that older adults would be affected by the primes to a greater extent than younger adults. Based on the research conducted by Brand and Schiebener [1], we predicted that there would be age differences in the GDT. Also, it was predicted that individual differences in personality and the DOSPERT would determine the degree to which participants were susceptible to the primes, because there has been research that has indicated that personality traits influence decision making in risky situations. We also predicted that there would be an interaction between prime type and risk propensity. That is, individuals with characteristics associated with a propensity to take risk might be more receptive to risk-taking primes than individuals without the propensity to take risks. Thus, we might observe more risk-taking on the GDT in the risk prime condition among individuals who have preexisting personality (*i.e.*, individuals who were extremely extraverted or extremely open to experience) characteristics associated with risk-taking. Lastly, we predicted that age differences in risk propensity would be attenuated after controlling for the effects of working memory.

## 2. Experimental Section

### 2.1. Participants

A total of 131 participants consisting of younger and older adults were analyzed in this study. Seventy-three younger adults (mean age = 19.55, SD = 2.45, range = 18–36) were recruited from the University of Alabama psychology subject pool. There were 38 males and 35 females. Fifty-eight older adults (mean age = 68.31, SD = 6.78, range = 60–83) were recruited from the surrounding communities in Tuscaloosa, Alabama and Thomasville, Georgia. There were 24 males and 34 females. Participants were randomly assigned to one of the three prime scenario conditions. This study was a 3 (Prime Scenario) × 2 (Age) between-subjects design. The random assignment to the various conditions was as follows: There were 20 older adults in the Risk-Seeking prime scenario condition, 20 older adults in the Risk-Aversive Condition and 18 older adults in the Control condition. With regard to the young adults, there were 44 young adults in the Risk-Seeing condition, 44 in the Risk-Aversive condition and 43 in the Control condition.

### 2.2. Materials

**Priming Scenarios**: As indicated earlier, we randomly assigned participants to one of three priming scenario conditions: Risk Seeking (RS), Risk-Aversion (RA), and Control. In the risk seeking scenario, the main character engaged in RS behavior and was rewarded for doing so. In the RA scenario, the main character avoided risk and was rewarded for his actions. Finally, in the Control condition, participants did extra math problems instead of reading a scenario. Thus, participants in the control condition were not exposed to any primes that should influence performance.

**WAIS-R**: The vocabulary subtest of the Wechsler Adult Intelligence Scale-Revised (WAIS-R) was used to test the vocabulary ability of each participant. This test was given to both younger and older adults to ensure that all participants had similar vocabulary abilities.

**Mini-Mental State Examination**: The Mini-Mental State Examination (MMSE) was given to the older adults participating in the experiment. The MMSE is a dementia screening instrument. According to the guidelines for scoring the MMSE, a score lower than 24 is indicative of dementia. Thus, older adults scoring less than 24 on the MMSE were not included in data analysis. As it turned out, no participants who agreed to be in the study scored below 24.

**Daneman and Carpenter Reading Span Task**: The Daneman and Carpenter Reading Span task was used to measure working memory. The automated reading span task that was used was created by Randy Engle and was accessed from the Inquisit Software website [36] (Unsworth, Heitz, Schrock, and Engle, 2005). The reliability for this task is 0.78 [37] (Conway, Kane, Bunting, Hambrick, Wilhelm, and Engle, 2005). Participants were presented with sentences followed by letters. The participants were instructed to determine if the sentence made sense or not. After all the sentences were presented, they were required to recall the letters presented for that trial in the correct order. The Daneman and Carpenter Reading Span task has been used to investigate performance on both language and non-language activities. This measure of working memory has been used to successfully predict performance in reasoning problems, playing bridge, controlling attention, and playing computer games [18].

**Game of Dice Task**: The Game of Dice Task was given to all participants on the computer to assess their willingness to take risk and make advantageous decisions. Participants were given the following instructions:

“Welcome to the Game of Dice Task. In this task, you are going to throw a virtual dice 18 times. Before each throw, you will be able to bet on the outcome by selecting a single number (e.g., ‘3’) or combinations of two to four numbers (e.g., ‘1-2-3’). You are given a starting capital of $1000. Your job is to maximize this capital within 18 throws of the dice. Good luck!”

The experimenter never mentioned the word “risk” nor were participants given information about the probabilities associated with the various presented options. Participants were shown all possible combinations from which they could choose.

In the long run, risky choices are associated with poorer outcomes. A “riskiness” score was calculated by taking the total number of risky choices and subtracting it from the total number of non-risky choices. Positive numbers (after the risk calculation) were an indication that participants made more safe choices than risky choices. In fact, the higher the positive number, the less risky the participants’ choices [38]. All information was visually presented to participants in each trial, including the following information: (a) amount of wins and losses; (b) current trials; (c) their current gain/loss for a specific trial; and (d) the total amount of money they have in their possession. Again, Figure 1 presents the initial scene that a participant would view upon beginning the task. Risk-taking in the GDT was assessed by subtracting the number of times participants chose risky options from the number of times they chose safe options. Options defined as risky were options from the top two rows (see Figure 1) and options defined as safe were options from the bottom two rows.

**The Big Five Personality Inventory**: The Big Five Inventory (BFI) is a 44 item inventory that measures an individual’s personality according to five major factors: (1) Extraversion; (2) Agreeableness; (3) Conscientiousness; (4) Neuroticism; and (5) Openness. Participants indicate how much they agree with different personality statements on a five point likert scale. One is “disagree strongly”, 3 is “neither agree nor disagree” and 5 is “agree strongly” [39].

The BFI is both a reliable and valid personality measure. The mean reliability score for the BFI is 0.83. Each of the personality dimensions of the Big Five Inventory also has high test-retest reliability. For extraversion it is 0.88, agreeableness is 0.79, conscientiousness is 0.82, neuroticism is 0.84, and openness is 0.81. The BFI has high scores of convergent validity when compared to Trait Descriptive Adjectives (TDA) and the Neuroticism-Extraversion-Openness Personality Inventory (NEO-PI). When comparing the BFI and the TDA the mean corrected pairwise convergent validity is 0.95. For the BFI and the NEO-PI it is 0.92 (John and Sirvastava, 1999) [40].

**Domain Specific Risk Attitude**: The Domain Specific Risk Attitude (DOSPERT) was given to all participants to assess their risk propensity in five different domains (ethical, financial, health/safety, recreational, and social). The DOSPERT also includes a scale assessing the individual’s perception of risk. For both subscales (risk-taking and risk perception), there were 30 questions with six questions in each domain. For the risk-taking subscale, participants were required to rate how likely they were to participate in such a risk. For the risk perception, participants were required to rate how risky they perceived each item to be. For the risk benefits, participants were required to rate the amount of benefits they thought they would obtain from those risks [41].

### 2.3. Procedure

Participants were tested independently in a quiet room. In the beginning, the experimenter gave participants a consent statement to read over. In addition, the experimenter reviewed the instructions with the participants and answered any questions that the participant had. Once the participant signed the consent statement and agreed to continue with the experiment, he/she was given a general demographic questionnaire to complete. Next, participants who were assigned to the RS and RA groups were presented with risk-seeking or a risk-aversive priming scenarios, respectively. As indicated earlier, participants in the neutral group did not receive a priming scenario, prior to the gambling task.

Participants in the risk-seeking and risk-aversive groups were told before reading the scenarios to pay close attention to the scenarios because they would be asked questions about them later. After the priming task, participants received several filler tasks. In the first filler task, participants received the questionnaire alluded to earlier. It asked them to rate the likability of the individual in the scenario and instructed them to write down as many adjectives as they could remember. The second filler task was math problems from a second grade workbook. Participants in the control group received an extra set of math problems instead of a priming scenario. There was a 10 to 15 min interval between the priming manipulation and the GDT.

Following the filler task, participants completed the GDT. Once the GDT was completed, participants were encouraged to take a break. After the break, participants received a reading span task, a test that measured working memory. Afterward, the reading span task the Need for Arousal scale, the BFI, DOSPERT, and the WAIS-R vocabulary test were completed. In addition, the MMSE was given to all older adult participants. The entire experiment took approximately one hour for younger adults and approximately one and a half to two h for older adults.

## 3. Results and Discussion

It should be noted that we will refer to two different indices of performance on the GDT: Risk-Taking and Money Total. The Risk-Taking score refers to the degree to which participants made risky choices. Money Total refers to how much money participants earned. We predicted that the prime presented before the GDT would influence the degree to which participants would be willing to take risks. We also predicted that personality and attitudes toward risk would both have a direct effect on Risk-Taking and that these variables could potentially moderate the effects of the prime. We conducted a series of analyses to follow-up on the aforementioned prediction. As indicated earlier, risk-taking on the GDT was assessed by subtracting the number of times participants chose risky options from the number of times they chose safe options. Options defined as risky were options from the top two rows (see Figure 1) and options defined as safe were options from the bottom two rows.

### 3.1. Prime and Risk-Taking

Table 1 presents the young and older adult data as a function of prime type. Keep in mind that the lower the number in each of the Prime Type columns, the riskier the choices. As one can see from reviewing Table 1, older adults show more risky behaviors than younger adults. However, there is not much difference in risk propensity among individuals within each of the age groups.

The above observations are supported by a 2 (age group *i.e.*, old *vs.* young) × (scenario *i.e.*, Risk-Seeking, Risk-Aversive, Control) analysis of variance (ANOVA) that was conducted to determine the degree to which age and priming scenarios affected Risk-Taking in the GDT. This analysis did not yield a significant main effect for scenario, *F*(1,125) = 0.39, *p* = 0.677. However, there was a significant main effect for age group, *F*(1,125) = 4.50, *p* = 0.036. Older adults (*M* = 13.69, *SD* = 15.16) were significantly more likely to take risks than younger adults (*M* = 18.77, *SD* = 12.45). In addition, there was no significant interaction between age group and scenario, *F*(2,125) = 0.678, *p* = 0.510.

**Table 1:** Age and scenario differences in risk scores.

Age Group	Risk-Seeking	Risk-Aversive	Control	Total
Young Adults	*M* = 16.50	*M* = 20.33	*M* = 19.44	*M* = 18.77
	*SD* = 12.29	*SD* = 13.02	*SD* = 12.24	*SD* = 12.45
Older Adults	*M* = 14.90	*M* = 15.00	*M* = 10.89	*M* = 13.69
	*SD* = 10.87	*SD* = 13.62	*SD* = 20.48	*SD* = 15.16
Total	*M* = 15.77	*M* = 17.91	*M* = 15.86	
	*SD* = 11.56	*SD* = 13.41	*SD* = 16.54	

Remember lower risk scores indicate riskier behavior.

### 3.2. Money Total on the GDT

In addition to conducting analyses to examine risk-taking on the GDT as a function of age and prime type, we conducted analyses to determine Money Total in the GDT. Money Total is determined by calculating how much money participants earn during the game. Theoretically, risk propensity (*i.e.*, risk-taking) and Money Total should be highly correlated because risky decisions are associated with high losses in the long run. Table 2 presents the data for Money Total as a function of age and prime type.

A 2 (age group *i.e.*, old *vs.* young) × 3 (scenario *i.e.*, Risk-Seeking, Risk-Aversive, Control) analysis of variance (ANOVA) was conducted to determine the degree to which age and priming scenarios affected money total in the GDT. This analysis did not yield a significant main effect for scenario, *F*(1,125) = 0.663, *p* = 0.517 or age group, *F*(1,125) = 1.485, *p* = 0.225. In addition, there was no significant interaction between age group and scenario, *F*(2,125) = 1.592, *p* = 0.208. Thus, the scenario (*i.e.*, the prime) had no effect on either risk-taking or Money Total on the GDT.

As indicated earlier, there were two indices of performance on the GDT: Money Total (*i.e.*, the amount of money earned) and Risk-Taking (*i.e.*, safe *vs.* risky choices). In previous studies, the risk-taking component has been considered most important. In the current study, we found age differences with respect to the risk-taking measure but no age differences with respect to Money Total on the GDT.

**Table 2:** Age and scenario differences in money total.

Age Group	Risk-Seeking	Risk-Aversive	Control	Total
Young Adults	*M* = −795.83	*M* = −416.67	*M* = −76.00	*M* = −424.66
	*SD* = 4531.58	*SD* = 3355.42	*SD* = 2708.52	*SD* = 3562.29
Older Adults	*M* = −800.00	*M* = −345.00	*M* = −2538.89	*M* = −1182.76
	*SD* = 3095.67	*SD* = 2832.24	*SD* = 5367.78	*SD* = 3916.64
Total	*M* = −797.73	*M* = −384.09	*M* = −1106.98	
	*SD* = 3901.07	*SD* = 3093.20	*SD* = 4167.24	

Moreover, the analyses indicated that the priming manipulation had no effect on Risk-Taking or Money Total in the GDT. However, we predicted that other factors, beside prime type, would influence risk-propensity with respect to the GDT. For example based on past studies [42,43], we predicted that certain personality traits would influence risk-taking.

### 3.3. Personality

We used the Big Five Personality Inventory to examine the effects of personality on the Risk-Taking measure of the GDT. Table 3 presents the degree to which Big Five personality traits are correlated as a function of age. As one can see, for the most part there were no significant age differences in personality traits except for conscientiousness (*p* = 0.01), with older adults being significantly more conscientious than younger adults. We were mainly interested in examining the degree to which extraversion and openness to experience predicted performance on the GDT due to the findings of previous research. For example, Nicholson and colleagues [23] administered questionnaires that assessed risk propensity to business students. In addition, he administered the BFI. He found that individuals who scored high on the extraversion and openness to experience dimensions were particularly prone to risky behavior.

Table 3 also provides correlations for personality traits, propensity to take risk, and Money Total. As one can see from Table 3, personality was not correlated with propensity to take risk (GDT) or with Money Total. However, we were interested in examining the possibility that the role of personality in predicting Risk-Taking and overall Money Total varied as a function of age group. Thus, we conducted separate analyses for each age group to determine if personality would be correlated with GDT Risk-Taking and Money Total for either or both of the age groups. Table 4 presents the data for just the younger adults. For younger adults, the analyses indicated that extraversion was significantly correlated with Risk-taking in the GDT, (*p* < 0.01). In fact, based on past research, we predicted that extraversion and openness to experience would be related to Risk-Taking in the GDT.

Table 5 presents the correlations for the older adults as a function of personality traits, risk propensity or risk-taking, and Money Total. As one can see, no personality traits were correlated with the risk propensity (*i.e.*, risk-taking) or the Money Total measures of performance for older adults.

In addition to being interested in the degree to which personality directly affects risk propensity (risk-taking) and Money Total in the GDT, we were also interested in examining the possibility that personality would moderate the degree to which the prime influenced risk-taking in the GDT.

We limited our moderation analyses to those personality dimensions that were hypothesized to predict risk-taking behavior and those personality traits that were shown to be significantly associated with risk-taking in our initial analyses. In our initial analysis with both age groups, there was no evidence that personality traits were significant moderators of the primes. However, because one variable in the younger adult data (*i.e.*, extraversion) significantly predicted risk-taking, we examined the possibility that extraversion would moderate the effects of the prime.

**Table 3:** Correlations between GDT, money total, age, working memory, and personality.

Measure	GDT	Money Total	Age	WM	Extraversion	Conscietousness	Openness	Agreeableness	Neuroticism
GDT	1	0.707 **	−0.195 *	0.264 **	−0.013	−0.152	−0.111	0.047	−0.093
Money Total		1	−0.098	0.212 *	−0.109	−0.156	−0.106	−0.077	−0.083
Age			1	−0.703 **	−0.198	0.345 **	0.104	0.124	0.070
WM				1	0.027	−0.236 **	−0.014	−0.141	−0.030
Extraversion					1	0.075	0.117	0.116	−0.252 **
Conscientiousness						1	0.168	0.453 **	−0.243 **
Openness							1	0.134	−0.124
Agreeable-ness								1	−0.464 **
Neuroticism									1

WM = Working Memory; Remember lower risk scores indicate riskier behavior; ** Correlation is significant at the 0.01 level (two-tailed); * Correlation is significant at the 0.05 level (one-tailed).

**Table 4:** Correlations between GDT, money total, working memory, and personality in young adults.

Measure	GDT	Money Total	WM	Extraversion	Conscietousness	Openness	Agreeableness	Neuroticism
GDT	1	0.763 **	0.279 *	−0.286 *	−0.010	−0.017	0.073	−0.176
Money Total		1	0.235 *	−0.269 *	−0.080	−0.063	0.026	−0.181
WM			1	−0.222	−0.018	0.007	−0.093	0.106
Extraversion				1	0.195	0.144	0.154	−0.232 *
Conscientiousness					1	0.009	0.424 **	−0.208
Openness						1	0.103	−0.108
Agreeable-ness							1	−0.460 **
Neuroticism								1

Remember lower risk scores indicate riskier behavior; ** Correlation is significant at the 0.01 level (two-tailed); * Correlation is significant at the 0.05 level (one-tailed).

**Table 5:** Correlations between GDT, money total, working memory, and personality in older adults.

Measure	GDT	Money Total	WM	Extraversion	Conscietousness	Openness	Agreeableness	Neuroticism
GDT	1	0.651 **	0.073	0.177	−0.204	−0.179	0.062	−0.023
Money Total		1	0.154	0.016	−0.198	−0.138	−0.176	0.048
WM			1	−0.051	0.020	0.212	−0.116	−0.075
Extraversion				1	0.104	0.132	0.125	−0.247
Conscientiousness					1	0.343 **	0.480 **	−0.414 **
Openness						1	0.156	−0.164
Agreeable-ness							1	−0.502 **
Neuroticism								1

Remember lower risk scores indicate riskier behavior; ** Correlation is significant at the 0.01 level (two-tailed); * Correlation is significant at the 0.05 level (one-tailed).

**Table 6:** Correlations between GDT, money total, age, working memory, and DOSPERT risk-taking.

Measure	GDT	Money Total	Age	WM	Ethical	Social	Finanical	Health/Safety	Recreational
GDT	1	0.707 **	−0.195 *	0.264 **	−0.105	0.127	−0.064	0.073	0.145
Money Total		1	−0.098	0.212 *	−0.080	0.110	−0.135	0.069	0.089
Age			1	−0.703 **	−0.483 **	−0.088	−0.373 **	−0.656 **	−0.581 **
WM				1	0.382 **	0.188 *	0.259 **	0.497 **	0.510 **
Ethical					1	0.313 **	0.380 **	0.623 **	0.332 **
Social						1	0.132	0.283 **	0.272 **
Financial							1	0.387 **	0.488 **
Health/Safety								1	0.635 **
Recreational									1

Remember lower risk scores indicate riskier behavior; ** Correlation is significant at the 0.01 level (two-tailed); * Correlation is significant at the 0.05 level (one-tailed).

#### Younger Adults and Moderation Analyses

**Figure 2:**
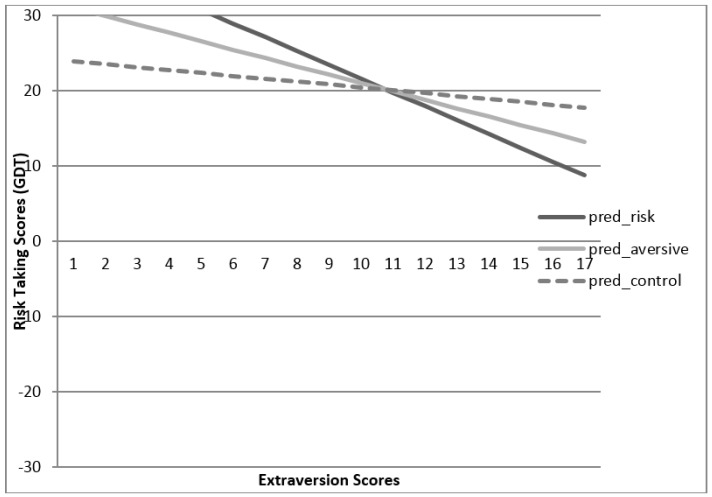
Extraversion as a moderator of prime & risk-taking scores in younger adults.

Multiple regression analyses were conducted to determine if personality moderated the strength of the prime in predicting risk-taking in the GDT. In this model, the main effects of prime and extraversion were included in the first two steps. Then a product variable, which was an interaction between extraversion and the prime, was included in the last step. In this model, both the main effects of prime and extraversion accounted for a significant amount of variance in risk scores, *R* = 5.8%, *F*(2,70) = 3.20, *p* = 0.047. The moderator variable, the interaction between prime and extraversion, was a marginally significant predictor of Risk-Taking in the GDT, *F*(3,69) = 2.54, *p* = 0.06. This model accounted for 6% of the variance in risk scores. Younger adults who received the risk seeking prime were riskier when their extraversion scores were higher *versus* when their extraversion scores were lower. Figure 2 illustrates the relation among extraversion scores on the BFI, prime condition, and risk-taking on the GDT. Specifically, individuals with high extraversion scores on the BFI were more apt to make risky decisions when receiving risk-related primes *versus* receiving neutral primes.

### 3.4. Dospert

As indicated earlier, the DOSPERT is an attitude and behavioral scale that is divided into three components: risk-taking, risk-perception, risk and ethics. For the risk-taking subscale, participants were presented with an event (*i.e.*, drag racing) and were instructed to rate the likelihood that they would participate in the described activity. Based on work by Rolison and colleagues [44], we initially predicted age differences in risk-taking with older adults being more cautious than young adults. Table 6 presents the data for the DOSPERT as a function of Age, Risk Propensity, and Working Memory, and the five DOSPERT scales. There are a couple of points to note from Table 6. Age is correlated with every DOSPERT domain, except the social one. This is because older adults self-reported being less risky than younger adults in every domain except for social. No DOSPERT domain was associated with risk-taking in the GDT or Money Total in the GDT. The primary take-home message with respect to the GDT is that older adults self-reported being less risky than younger adults in every domain, except the social domain.

Thus, our hypothesis that older adults would be more susceptible to the prime was not confirmed. If anything, a certain subset of younger adults who scored high on extraversion were marginally more susceptible to the prime than younger adults who didn’t score as high on extraversion on the BFI.

### 3.5. Working Memory

We predicted that the source of age differences in risk-taking in the GDT would be working memory. Thus, we predicted that the relation between age and risk-taking in the GDT would be reduced after controlling for working memory. Using Baron and Kenny’s [45] analysis, first we used regression analyses to indicate that age was a significant predictor of Risk-Taking, *F*(1,129) = 5.095, *p* = 0.026, *β* = −0.109, *p* = 0.026. Next, we demonstrated that the mediator variable, working memory, was a significant predictor of Risk-Taking, *F*(1,129) = 9.65, *p* = 0.002, *β* = 0.171, *p* = 0.002. Next, to test for a partial mediation, both age and working memory were included in the analysis to predict Risk-Taking. This analysis demonstrated that if age were entered first into the equation, and working memory were entered second, working memory was a significant predictor, *F*(2,128) = 4.80, *p* = 0.01, *β* = 0.163, *p* = 0.038 of Risk-Taking. However if working memory were entered first into the equation, age was no longer significant in explaining the variance after controlling for working memory, *β* = −0.01, *p* = 0.876. Thus, it appears that the relation between Age and risk-taking was mediated through working memory.

## 4. Discussion

### 4.1. Game of Dice Task (GDT)

We conducted this study to replicate and follow-up on research conducted by Brand and colleagues [1,4]. Brand and Schiebener [1] examined age differences on the GDT and found that older adults were riskier than younger adults, and that younger adults had superior performance as measured by Money Total. Like Brand and Schiebener, we found age differences in risk-taking. We also conducted mediational analyses that yielded evidence that the age differences were mediated through executive/working memory resources. In addition to replicating Brand and Schiebener’s work, we extended their work by presenting a prime prior to administering the gambling task and by examining the degree to which personality and preexisting attitudes affected performance on the GDT. We will expound upon each of the aforementioned issues. We will first focus on the way in which working memory affected performance on the GDT.

### 4.2. GDT and Working Memory

One of the interesting aspects of this study is that it measures individuals’ propensity to take risk when Risk-Taking is not rewarded. However, we would argue that it takes working memory resources to realize that Risk-Taking will not be rewarded on the GDT and to calculate the actual risks associated with each of the options. As indicated earlier, when participants receive the standard instructions, they are not explicitly told that risk-taking will result in suboptimal performance. They are simply told that they will be able to bet outcomes based on a single number or based on dice combinations. Upon reading the instructions, it is assumed that participants will make the inference that betting on grouped combinations of dice will be less risky than betting on a single number. However, making such an inference requires working memory resources [8,9,46,47].

In fact, there are two ways that participants could produce optimal performance on the GDT: through calculating probabilities based on the instructions or through feedback [1,3,5] upon playing the game. To fully understand the probabilities associated with choices, participants would have to engage in some level of computations and there is evidence that numeracy is associated with working memory resources [10,19,46,47,48].

Another way that participants could develop a successful strategy for optimizing performance is by adjusting their strategy based on feedback. However, the ability to adjust strategy would take working memory/executive processing to monitor outcomes of choices; to notice patterns; and—to develop a strategy based on observed patterns [1].

As it turned out, working memory predicted risk-taking on the GDT more than any other variable. In fact, working memory fully mediated the relation between age and risk-taking on the GDT. That is, age per se did not matter after controlling for working memory. It just so happened that age was correlated with working memory. Thus, older adults with sufficient working memory resources were as capable of avoiding risky choices as younger adults, with respect to the GDT.

### 4.3. Working Memory and Age Differences in the GDT

One of the most robust findings in the cognitive aging literature is that working memory declines as a function of age [4,49]. Thus, it was not surprising that the current gambling task yielded significant age differences, given that risk-taking on the GDT was so reliant on working memory. However, we would argue that one way that we were able to extend work by Brand and colleagues [1,3,5] was by conducting mediational analyses to determine the extent to which working memory mediated the relation between age and risk-taking on the GDT. In this study, cognitive resources not only moderated the degree to which age predicted risk-taking on the GDT, but cognitive resources (*i.e.*, working memory) actually mediated the relation between age and risk-taking.

We chose the Daneman and Carpenter Reading Span Task as our measure of working memory, because it measures a variety of cognitive functions associated with working memory. Although heretofore, researchers have emphasized the relation between the GDT and task-switching, we would argue that the GDT involves many more cognitive functions. For example, the GDT requires participants to make inferences to fully understand the level of risk associated with the various options. The ability to make inferences based on written and oral messages is highly tied to working memory [9,19,46,50,51]. Moreover, optimal performance on the GDT, with respect to risk-taking and Money Total, is associated with the ability to develop a strategy. The ability to develop a strategy is highly dependent on working memory and executive processes [47,52] and we believe that learning from feedback involves executive processing and resources typically associated with working memory. What is interesting about the results is that they indicate that there is no age difference in decision-making as long as young and older adults have comparable working memory resources.

However, there is another issue that needs to be addressed with respect to age and risk-taking on the GDT: the issue of motivation. There is evidence that older adults can be cognitive misers. That is, according to Craik and Byrd [21], older adults are less likely than younger adults to use self-initiated strategies to optimize performance, in part because cognitive resources decline with age and it is more difficult for older adults to spontaneously use strategies than it is for younger adults. Thus, with regard to risk-taking and Money Total on the GDT, older adults may be less motivated than younger adults to think of strategies to optimize performance.

Hess and colleagues explain older adults’ reluctance to use strategies via Balte’s Selective Optimization model [53]. Due to age-related changes in cognitive efficiency, older adults are selective about the expenditure of cognitive resources [54,55,56]. In a number of studies, Hess and colleagues have shown that when a decision is complex and requires extensive cognitive processing, older adults often use a heuristic rather than engage in effortful processing, especially if the consequences of the decision are not relevant to them and they will not be held accountable for their decisions [57]. However, when the same task is changed so that older adults will be held accountable for their decisions, their performance improves—and, in many instances, the age difference disappears. Other researchers investigating age differences in decision-making have found similar results to that of Hess and colleagues [58].

Thus, in many of these instances older adults are capable of using effortful processing to optimize performance, but will not engage in this effortful processing unless they will be required to be accountable for their judgments or performance. It is possible that older adults were capable of performing as well as younger adults, but that older adults were not motivated to expend the energy to determine the probabilities associated with various outcomes or to encode the instructions well enough to appreciate the level of risk associated with their choices. Likewise, older adults may not have been motivated to monitor feedback to change their behavior.

Recently, decision-making researchers have become increasingly aware that decision-making doesn’t occur in a vacuum and issues such as motivation need to be considered in order to predict decision-making outcomes [54,55,56,59]. In addition, researchers have become aware that individual differences in personality can affect the way in which individuals approach decision making.

### 4.4. Personality and the GDT

There have been studies that have been conducted that have shown that variables such as impulsiveness, perfectionism, and neuroticism affect the propensity to take risk on the GDT [24,60]. We examined the effect of personality on Risk-Taking in the current study by administering the Big Five Personality Inventory to participants. When examining personality across all age groups, we found that there were no personality traits that were significant predictors of risk propensity on the GDT. However, when age groups were examined separately, extraversion was found to be a significant predictors of Risk-Taking on the GDT for younger but not older adults. No personality traits were significant predictors of Risk-Taking on the GDT for older adults. This might be due to the older adults not fully appreciating the consequences of their decisions based on the instructions and feedback. Thus, an older adult who is very cautious may have made risky decisions even though she normally would not have made those choices had she understood the consequences of her choices.

### 4.5. Priming Scenario

In addition to research indicating that personality can affect risky decision-making, there is evidence that contextual variables can affect risk. Thus, individuals who are exposed to information emphasizing loss due to risk-taking (e.g., losing money due to gambling at a casino) might be more cautious than individuals not exposed to that same type of information. However, in the current study, there was no evidence that the prime had an effect when the young and older adult data was analyzed together.

Interestingly, there was evidence that the priming manipulation was having an effect on the young adults’ risk-taking propensity when the two age groups were analyzed separately. That is, for young adults the data was in the predicted direction as a function of priming condition; younger adults were more risky when the gambling task was preceded by a prime than when the gambling task was not. However, the priming effects failed to reach significance.

One reason that there were not greater effects as a function of prime condition is that the prime manipulation may not have been strong enough. In a meta-analysis, researchers [61] found that risk glorifying content such as video games, advertisements, and film had greater effects than verbal stimuli in glorifying risk. In fact, they found that video games had the greatest effect, followed by advertisements and then film and television. It is this issue that we turn to next.

### 4.6. Moderation Effects in Prime Susceptibility

We initially predicted that personality traits and individual characteristics, such as scores on the BFI, would moderate how susceptible participants would be to the prime. Based on past research, we hypothesized that participants high in extraversion, openness, and neuroticism would be more susceptible to the risk-seeking primes [23,42,62] than individuals low on the aforementioned traits.

When we analyzed the older and younger adult personality data together, there was no evidence that personality traits moderated the effects of the prime. However, when we examined the two age groups separately, there was evidence that younger adult high in extraversion were more susceptible to the effects of the risk-taking prime than younger adults who were low in extraversion. We concede that the marginally significant finding with respect to extraversion and the risk-seeking prime must be viewed with some caution, as only one of the personality traits was marginally significant and its significance was based on our analysis of just the younger adult data. However, it gives some credence to the notion that contextual variables have the potential to influence risk-taking on the GDT.

## 5. Conclusions

In conclusion, we acknowledge that this research has several limitations. First, a number of analyses were conducted on the data which increases the probability of committing a Type I error; thus, we acknowledge that marginal effects (such as the finding that extraversion moderated the effects of the risk-seeking prime) should be treated with some caution. Also, we acknowledge that the age differences reported in this study, may be due in part to motivation.

Nevertheless, we believe that the current study has important implications for the real world. This study, along with other studies focusing on decision-making, provides evidence that contextual variables and preexisting personality characteristics may influence decision making—at least for younger adults. We would argue that older adults may not have fully understood the consequences of their choices. As a result, we were not able to fully assess the impact of personality on decision making, for individuals in late adulthood.

One of the most intriguing findings is that older adults self-reported being more cautious than younger adults on the DOSPERT, but nevertheless made more risky choices than younger adults. The DOSPERT indicates that when older adults fully appreciate the level of risk associated with their choices, they would be less risky rather than more risky than younger adults. Thus, we would argue that the age differences may be due, in large part, to older adults not fully appreciating the level of risk associated with their decisions. We believe that these data have implications for situations in which older adults have to make complex decisions but donot fully appreciate the level of risk associated with their choices. Older adults may unwittingly enter into risky agreements involving insurance plans or agreements involving reversed mortgages that could result in their losing a substantial amount of money. Thus, the issue of age and risky decision-making needs to be explored in more depth.
